# Effect of IRT5 probiotics on dry eye in the experimental dry eye mouse model

**DOI:** 10.1371/journal.pone.0243176

**Published:** 2020-12-01

**Authors:** Jayoon Moon, Jin Suk Ryu, Jun Yeop Kim, Sin-Hyeog Im, Mee Kum Kim

**Affiliations:** 1 Department of Ophthalmology, Seoul National University College of Medicine, Seoul, Republic of Korea; 2 Laboratory of Ocular Regenerative Medicine and Immunology, Seoul Artificial Eye Center, Seoul National University Hospital Biomedical Research Institute, Seoul, Republic of Korea; 3 Division of Integrative Biosciences and Biotechnology, Department of Life Science, Pohang University of Science and Technology, Pohang, Republic of Korea; 4 ImmunoBiome Inc. POSTECH Biotech Center, Pohang, Republic of Korea; McMaster University, CANADA

## Abstract

**Objective:**

To investigate the clinical effects of IRT5 probiotics in the environmental dry eye model.

**Methods:**

Eight week old male C57BL/6 mice were randomly divided into two groups; control group (n = 16) received oral gavage of 300 μL phosphate-buffered saline (PBS) alone once daily, IRT5 group (n = 9) received oral gavage of 1 x 10^9^ CFU IRT5 probiotics powder in 300 μL PBS once daily, both groups for 11 to 12 days. Simultaneously, all mice underwent dry eye induction. Tear secretion, corneal staining and conjunctival goblet cell density were evaluated. Quantative real-time polymerase chain reaction (RT-PCR) for inflammation-related markers was performed. 16S ribosomal RNA of fecal microbiome was analyzed and compositional difference, alpha and beta diversities were assessed.

**Results:**

There was no difference in NEI score but significant increase in tear secretion was observed in IRT5 group (p < 0.001). There was no significant difference in goblet cell density between groups. Quantative RT-PCR of cornea and conjunctiva revealed increased TNF-α expression in IRT5 group (p < 0.001) whereas other markers did not significantly differ from control. IRT5 group had significantly increased species diversity by Shannon index (p = 0.041). Beta diversity of genus by UniFrac principle coordinates analysis showed significant distance between groups (p = 0.001). Compositional differences between groups were observed and some were significantly associated with tear secretion. Multivariate linear regression analysis revealed *Christensenellaceae* (p = 0.009), *Lactobacillus Helveticus group* (p = 0.002) and *PAC001797_s* (p = 0.011) to strongly influence tear secretion.

**Conclusion:**

In experimental dry eye model, IRT5 probiotics treatment partially improves experimental dry eye by increasing tear secretion which was associated with and influenced by the change in intestinal microbiome. Also, intestinal microbiome may affect the lacrimal gland through a different mechanism other than regulating inflammation.

## Introduction

Dry eye disease is mainly caused by the evaporative water loss which consequently leads to hyperosmolar tissue damage [1]. In other words, this decrease in ocular surface wettability results in the entrance to a vicious cycle of early tear film breakup and aggravated hyperosmolarity [1]. Several experimental dry eye disease studies have identified its association with immune responses on the ocular surface, such as T helper 17 (Th17) cells and their inflammation-related cytokines, antigen presenting cells and inflammatory M1 phenotype macrophages [2–6]. Therefore, the main underlying mechanism for dry eye disease is the autoimmune based inflammatory reaction taken place on the ocular surface [5, 7].

Over the past decade, intestinal microbiome’s importance in indirectly affecting both local and systemic immunity has emerged and numerous studies have been performed to identify its relation to disease and health [8–10]. Particularly, dysbiotic intestinal microbiome has been reported to be associated with and to indirectly affect several autoimmune diseases, such as Sjögren’s syndrome and inflammatory bowel disease [11–14]. Moreover, dysbiotic intestinal microbiome has been observed to affect ocular manifestations of autoimmune diseases in both experimental models and clinical subjects [11, 15, 16]. In our previous clinical study, we observed significant differences in intestinal microbiome of Sjögren’s syndrome patients compared to normal subjects and that this dysbiosis was associated with dry eye severity [17]. Interestingly, through this study, we also noticed that the intestinal microbiome of ordinary environmental dry eye subjects displayed characteristics somewhere in between Sjögren’s syndrome and normal subjects [17]. Immune responses to inflammatory reaction of dry eye disease may also be linked to intestinal microbiome [16].

Since dysbiotic intestinal microbiome has shown to influence immunity, there have been several studies using probiotics or fecal transplantation to see if altering or normalizing intestinal microbiome can affect clinical manifestations of various diseases and health, and promising results had been observed [18–22]. IRT5 probiotics, a mixture of *Bifidobacterium bifidum*, *Lactobaccillus acidophilus*, *Lactobacillus casei*, *Lactobacillus reuteri* and *Streptococcus thermophilus*, was reported to exert anti-inflammatory effects in several autoimmune models [20, 22, 23]. We have also demonstrated IRT5 probiotics’ beneficial effects of alleviating clinical manifestations in autoimmune uveitis and autoimmune dry eye models [24]. More recently, we observed that IRT5 probiotics acts via downregulation of antigen-presenting processes in the immune network [25].

Standardized treatment protocols for autoimmune or environmental dry eye syndrome have been established and are being constantly updated [26]. However, there are some patients with insufficient symptom and/or sign relief despite extensive topical medications and environment modifications. Therefore, identifying a supplementary or additive treatment option that can aid in fully alleviating these unmet needs may help better control dry eye syndrome. Herein, we investigate the clinical effects of IRT5 probiotics in the environmental dry eye model.

## Materials and methods

### Animal

All mice were managed in accordance with the Association for Research in Vision and Ophthalmology guidelines for the Use of Animals in Ophthalmic and Vision Research. The study protocol was approved by the Institutional Animal Care and Use Committee of the Seoul National University Biomedical Research Institute (IAUCUC No. 18-0129-S1A0 and 19-0076-S1A0). All examination and sacrifice were performed under anesthesia by intramuscular injection of a mixture of zoletil (30 mg/kg) and xylazine (10 mg/kg). All efforts were made to minimize suffering.

Eight week old male C57BL/6 mice (Koatech, Gyungi-Do, Republic of Korea) were used. All mice (n = 25) were bred under a specific pathogen-free environment and maintained at 22–24°C, relative humidity 55% ± 5%, with free access to water and food at the Mouse Facility at Biomedical Research Institute of Seoul National University Hospital (Seoul, Republic of Korea). Excretory feces in each cage were collected, minced, mixed together and re-distributed to all cages, so that all mice shared their excretory feces in the cages to simulate co-housing. Overall health was monitored twice a week (weight and hair loss). Mice were randomly divided into two groups; control group (n = 16) received oral gavage of 300 μL phosphate-buffered saline (PBS) alone once daily, IRT5 group (n = 9) received oral gavage of 1 x 10^9^ CFU IRT5 probiotics powder in 300 μL PBS once daily, both groups for 11 to 12 days. Simultaneously, all mice underwent dry eye induction. At the end of the experiment, euthanasia was performed using compressed CO_2_ gas, according to the American Veterinary Medical Association Guidelines for the Euthanasia of Animals: 2013 Edition.

### Environmental dry eye induction

**Fig 1:**
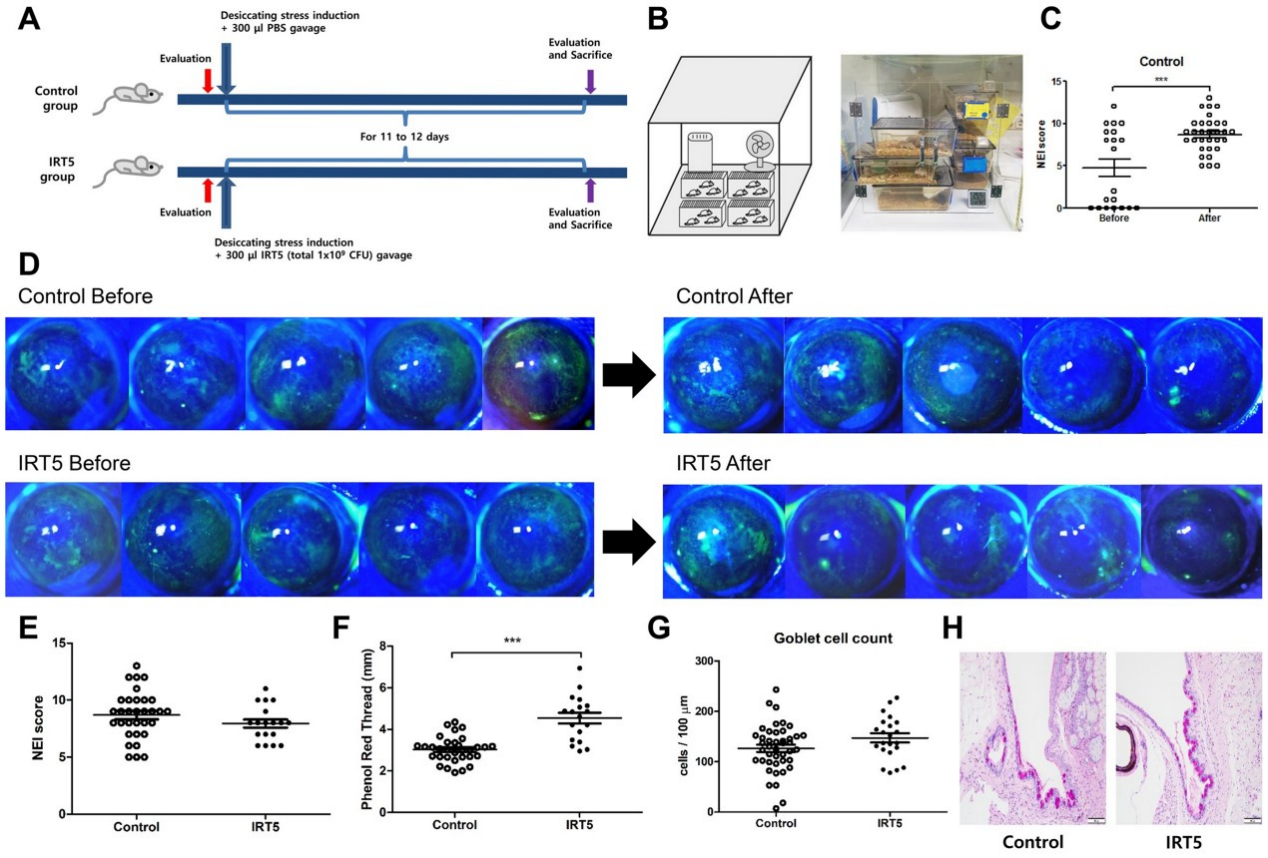
Environmental dry eye induction design and clinical results. Eight-week old male C57BL/6 mice were divided into control and IRT5 groups and underwent desiccating stress induction with oral gavage of either 300 μL PBS or 1 x 10^9^ CFU IRT5 probiotics powder in 300 μL PBS, both groups for 11 to 12 days (A). Simultaneously, all mice were exposed to drafty low humid (30–35%) environment all day for 11 to 12 days (B) with sterile intraperitoneal injection of scopolamine hydrobromide (Sigma, Saint Louis, USA) (0.5 mg / 0.2 ml) three times a day. Significant NEI score increase in the control group indicated appropriate desiccating stress induction (p < 0.001) (C). Representative corneal fluorescein stained photos of 5 mice in each group are shown in D. There was no difference in NEI score but significant increase in tear secretion was observed in IRT5 group (p < 0.001) (E and F). There was no difference in goblet cell density between groups, though IRT5 group showed a slight increase (p = 0.103) (G and H). NEI score: National Eye Institute score. Statistical analysis with error bars indicating mean and SEM of data points by Mann–Whitney U test: *** p < 0.001.

Desiccating stress for dry eye induction was done to eight-week old male C57BL/6 mice by sterile intraperitoneal injection of scopolamine hydrobromide (Sigma, Saint Louis, USA) (0.5 mg / 0.2 ml) three times a day and keeping them exposed to an environment with drafty low humidity (30–35%) all day for 11 to 12 days (Fig 1A and 1B).

### Preparation of IRT5 probiotics mixture

IRT5 probiotics power (1 × 10^9^ CFU/g), which consists *L*. *casei*, *L*. *acidophilus*, *L*. *reuteri*, *B*. *bifidum*, and *S*. *thermophiles* and contains 2 × 10^8^ CFU/g of each strain, was kindly provided by Young-Tae Ahn (Korea Yakult Co., Giheung, South Korea).

### Clinical evaluation

Tear secretion was evaluated with phenol red-impregnated cotton threads (FCI Ophthalmics, Pembroke, MA, USA) which were inserted into the lateral canthus of anesthetized (anesthesia by using a mixture of zoletil and xylazine at a ratio of 1: 3) mice for 60 seconds. The wet length of the wet thread was measured in millimeters.

Corneal staining evaluation was performed by instilling one drop of 0.5% Fluorescein to the lower lateral conjunctival sac and observing under cobalt light excitation. Corneal epithelial defect was scored in a blind manner by one investigator (JM) using National Eye Institute score (NEI score) [27].

### Conjunctival goblet cell assessment

The conjunctiva was excised and fixed in 10% formalin. The samples were sliced and stained using PAS staining kit according to manufacturer’s instruction. Mucin-filled goblets cells were observed and counted in a blind manner by 3 investigators (JM, JSR and JYK), and were averaged for analysis based on the protocol in previous study [28, 29].

### Quantative real-time polymerase chain reaction

The cornea and conjunctiva, and extraorbital lacrimal gland were cut into small pieces and lysed in RNA isolation reagent. After sonication with a probe sonicator (Ultrasonic Processor, Cole Parmer Instruments, Vernon Hills, IL), total RNA was extracted using RNeasy Mini kit (Qiagen, Venlo, Netherlands), and first‐strand cDNA was synthesized by reverse transcription (High Capacity RNA‐to‐cDNA Kit, Applied Biosystems, Foster City, CA). Real‐time amplification was performed by TaqMan Universal polymerase chain reaction (PCR) Master Mix (Applied Biosystems) in an automated instrument (ABI 7500 Real Time PCR System, Applied Biosystems) targeting tumor necrosis factor (TNF)-α (Mm00443258_m1, Thermo fisher, Waltham, USA), interferon (IFN)-γ (Mm01168134_m1, Thermo fisher, Waltham, USA), interleukin (IL)-1β (Mm00434228_m1, Thermo fisher, Waltham, USA), IL-6 (Mm00446190_m1, Thermo fisher, Waltham, USA), IL-17A (Mm00439618_m1, Thermo fisher, Waltham, USA), IL-8 (Mm04207460_m1, Thermo fisher, Waltham, USA), IL-10 (Mm00439614_m1, Thermo fisher, Waltham, USA), matrix metallopeptidase-9 (MMP-9, Mm00442991_m1, Thermo fisher, Waltham, USA) for cornea and conjunctiva, and TNF-α (Mm00443258_m1, Thermo fisher, Waltham, USA), IFN-γ (Mm01168134_m1, Thermo fisher, Waltham, USA), IL-1β (Mm00434228_m1, Thermo fisher, Waltham, USA), IL-17A (Mm00439618_m1, Thermo fisher, Waltham, USA), class II major histocompatibility complex (MHC-II, Mm00439216_m1, Thermo fisher, Waltham, USA), B cell activating factor (BAFF, Mm00446347_m1, Thermo fisher, Waltham, USA) for extraorbital lacrimal gland.

### Fecal microbiota 16S ribosomal RNA analysis

Fecal pellets were collected at the beginning and end of study. They were directly collected from the anus of each mouse by holding it and allowing defecation. The collected feces were immediately stored at -80°C till analysis. Fecal samples were referred to Chunlab, Inc. (Seoul, Republic of Korea) for analysis. V3 to V4 region of 16S ribosomal RNA (rRNA) analysis was performed at Chunlab, Inc. (Seoul, Republic of Korea) in the same way as described in our previous study.[25] Compositional differences, alpha and beta diversities (using Unifrac), and linear discriminant analysis (LDA) effect size (LEfSe) of intestinal microbiome were evaluated. Only those taxa that showed a p value < 0.05 and a log LDA score ≥ 2 were ultimately considered for biomarker evaluation.

### Statistical analysis

Statistical analyses were performed using SPSS software version 22 (SPSS, Inc, Chicago, IL) and GraphPad software version 5 (GraphPad Software, San Diego, CA). Outliers were excluded and Mann–Whitney U test was performed for comparison between groups. Comparison of intestinal microbiome composition among groups was done with Wilcoxon rank-sum test. Univariate and multivariate linear regression analysis were performed to determine relation between clinical signs and intestinal microbiome. The family and species variables with p < 0.2 observed in univariate linear regression analysis were included in multivariate linear regression analysis to assure all pertinent and potential predictive variables. P values less than 0.05 were accepted as statistically significant. The results are presented as mean ± SEM unless otherwise indicated.

## Results

### IRT5 probiotics treatment improves tear secretion in environmental dry eye model

Changes in corneal fluorescein staining was seen in both groups. Significant increase in NEI score of control group was observed indicating that environmental dry eye was successfully induced (p < 0.001, Fig 1C and 1D). There was no difference in NEI score but significant increase in tear secretion was observed in IRT5 group (p < 0.001, Fig 1E and 1F). There was no significance in goblet cell density between groups, though IRT5 group showed a slight increase (p = 0.103, Fig 1G and 1H).

### IRT5 probiotics treatment increases TNF-α expression in cornea and conjunctiva

**Fig 2:**
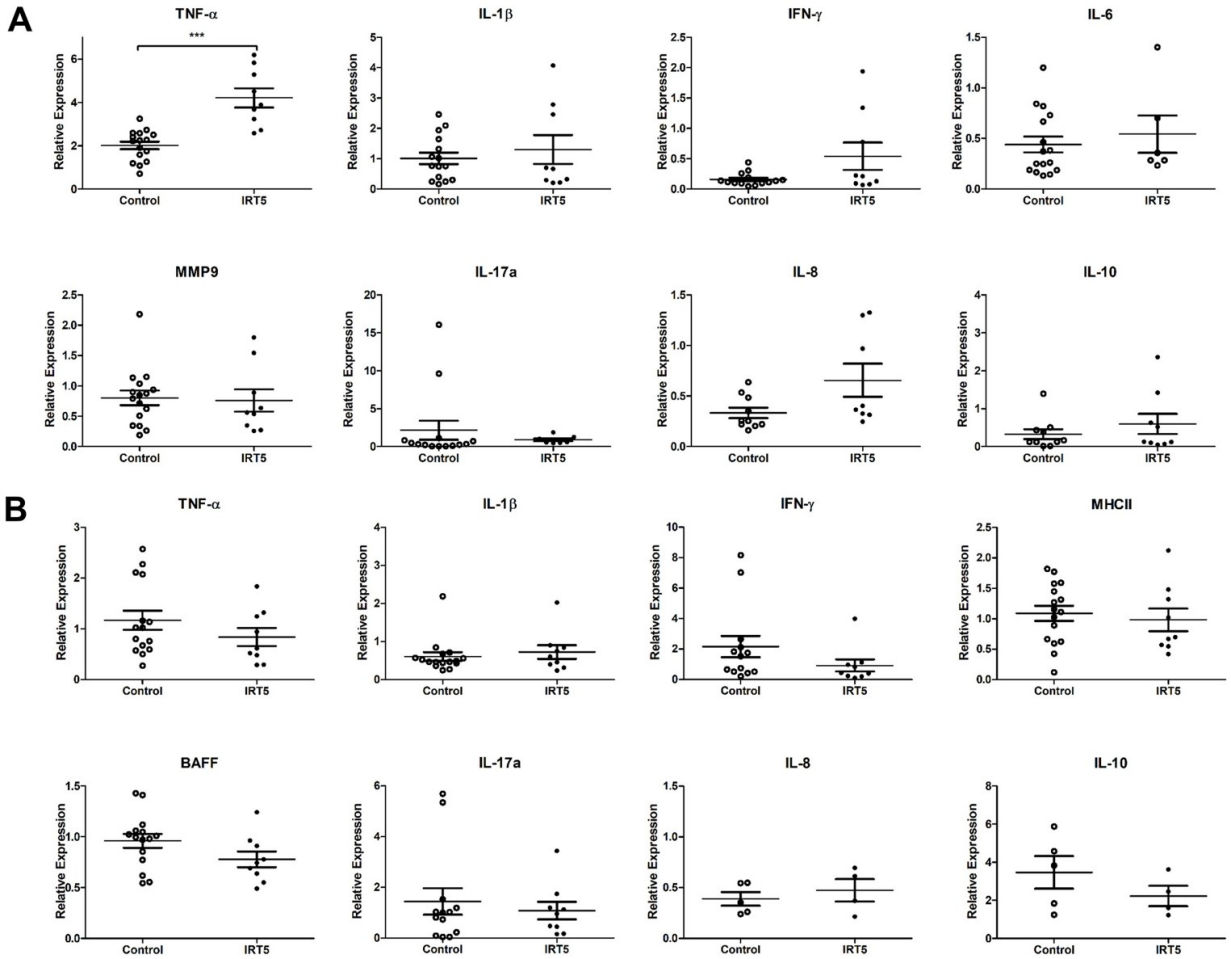
Quantative real-time PCR of cornea and conjunctiva, and extraorbital lacrimal gland. Quantative real-time PCR of inflammatory markers’ RNA transcripts was performed in cornea and conjunctiva (A), and extraorbital lacrimal gland (B). The results of quantative real-time PCR of cornea and conjunctiva are shown in A. It revealed increased TNF-α expression in IRT5 group (p < 0.001) whereas other markers did not significantly differ from control (A). The results of quantative real-time PCR of extraorbital lacrimal gland are shown in B. It did not show any significant differences in all inflammatory markers (p > 0.05) (B). Statistical analysis with error bars indicating mean and SEM of data points by Mann–Whitney U test: *** p < 0.001.

Quantative real-time PCR of cornea and conjunctiva revealed increased TNF-α expression in IRT5 group whereas other markers did not significantly differ from control (p < 0.001, Fig 2A). Extraorbital lacrimal gland did not show significant differences in all inflammatory markers (Fig 2B).

### IRT5 probiotics treatment alters intestinal microbiome composition

**Fig 3:**
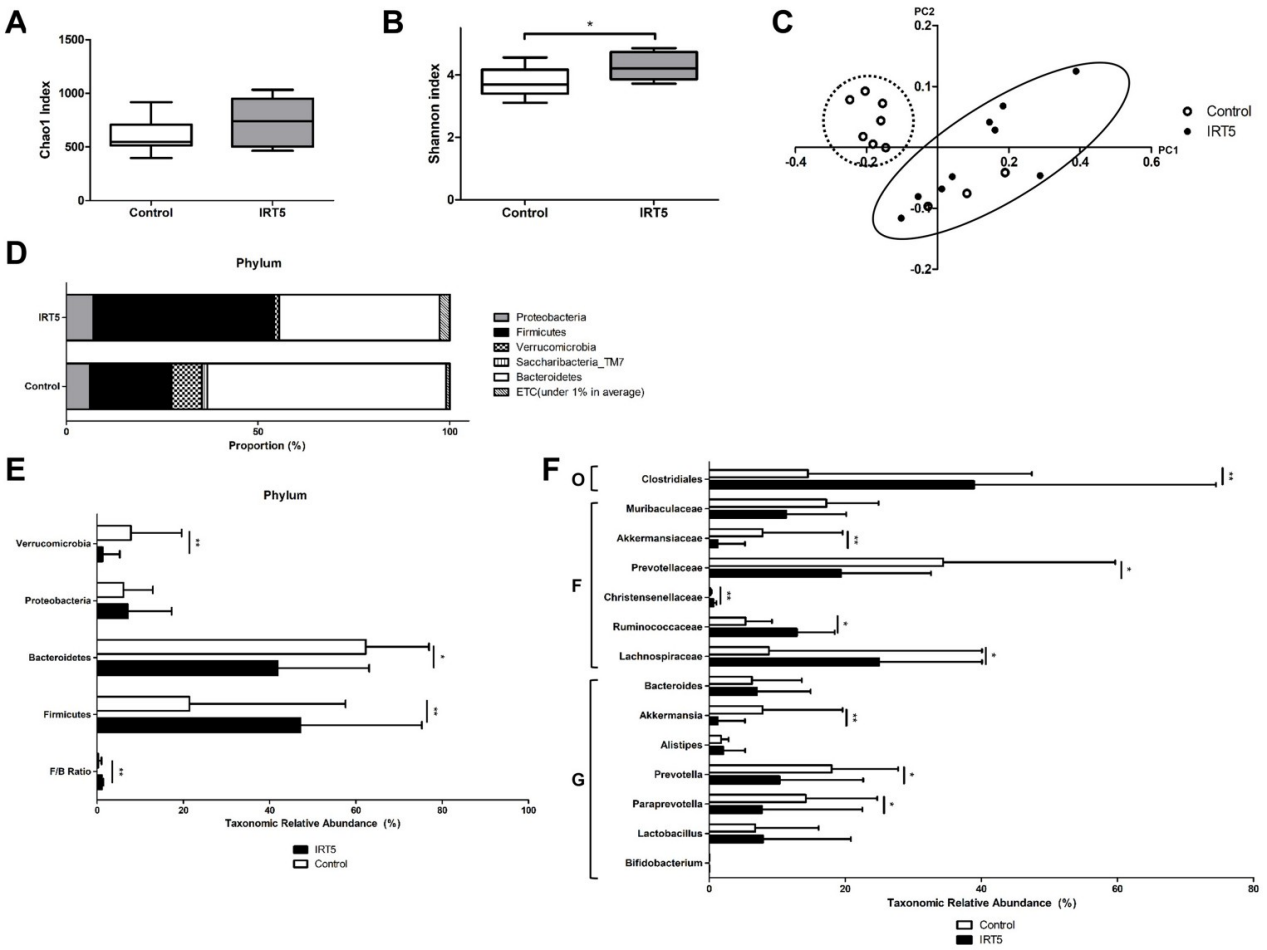
Intestinal microbime compositional changes, alpha and beta diversity. There was no difference in species richness by Chao1 index (p > 0.05) (A). However, significant increase in species diversity by Shannon index was observed in IRT5 group (p = 0.041) (B). Beta diversity of genus by UniFrac principle coordinates analysis showed significant distance between groups (p = 0.001) (C). Compositional differences in phylum between groups were observed, such as *Verrucomicrobia* (p = 0.009), *Bacteroidetes* (p = 0.011), *Firmicutes* (p = 0.009) (D and E). The *Firmicutes* / *Bacteroidetes* (F/B) ratio significantly increased in the IRT5 group (p = 0.009) (E). In class, IRT5 group showed increased *Clostridiales* (p = 0.009) (F). In family, IRT5 group had decreased *Akkermansiaceae* (p = 0.009) and *Prevotellaceae* (p = 0.014), and increased *Christensenellaceae* (p = 0.001), *Ruminococcaceae* (p = 0.018), *Lachnospiraceae* (p = 0.018) (F). In genus, IRT5 group revealed to have decreased *Akkermansia* (p = 0.009), *Prevotella* (p = 0.041) and *Paraprevotella* (p = 0.041) (F). There was no significant difference in *Lactobacillus* and *Bifidobacterium* between groups (F). O: order, F: family, G: genus. Statistical analysis with error bars indicating the minimum and maximum data points by Wilcoxon rank-sum test: * p < 0.05, ** p < 0.01.

There was no difference in species richness (Fig 3A) while significant increase in species diversity by Shannon index (p = 0.041, Fig 3B) was observed. Beta diversity of genus by UniFrac principle coordinates analysis showed significant distance between groups (p = 0.001, Fig 3C). Compositional differences in phylum between groups were observed, such as *Verrucomicrobia*, *Bacteroidetes*, *Firmicutes* (p < 0.05, Fig 3D and 3E). The *Firmicutes* / *Bacteroidetes* (F/B) ratio significantly increased in the IRT5 group (p < 0.01, Fig 3E). In class, IRT5 group showed increased *Clostridiales* (p = 0.009, Fig 3F). In family, IRT5 group had decreased *Akkermansiaceae* (p = 0.009) and *Prevotellaceae* (p = 0.014), and increased *Christensenellaceae* (p = 0.001), *Ruminococcaceae* (p = 0.018), *Lachnospiraceae* (p = 0.018) (Fig 3F). In genus, IRT5 group revealed to have decreased *Akkermansia* (p = 0.009), *Prevotella* (p = 0.041) and *Paraprevotella* (p = 0.041) (Fig 3F). There was no significant difference in *Lactobacillus* and *Bifidobacterium* between groups (Fig 3F).

LEfSE analysis revealed significant biological taxonomic differences between groups. A total of 159 operational taxonomic units (OTUs) differed between groups (S1 Table). Among them, 110 OTUs were more abundant and 49 OTUs were scarce in IRT5 group compared to control (S1 Table). Also, 86 OTUs were species of which 61 OTUs were more abundant and 25 OTUs were scarce in IRT5 group (S1 Table).

### Tear secretion is associated with intestinal microbiome change

**Fig 4:**
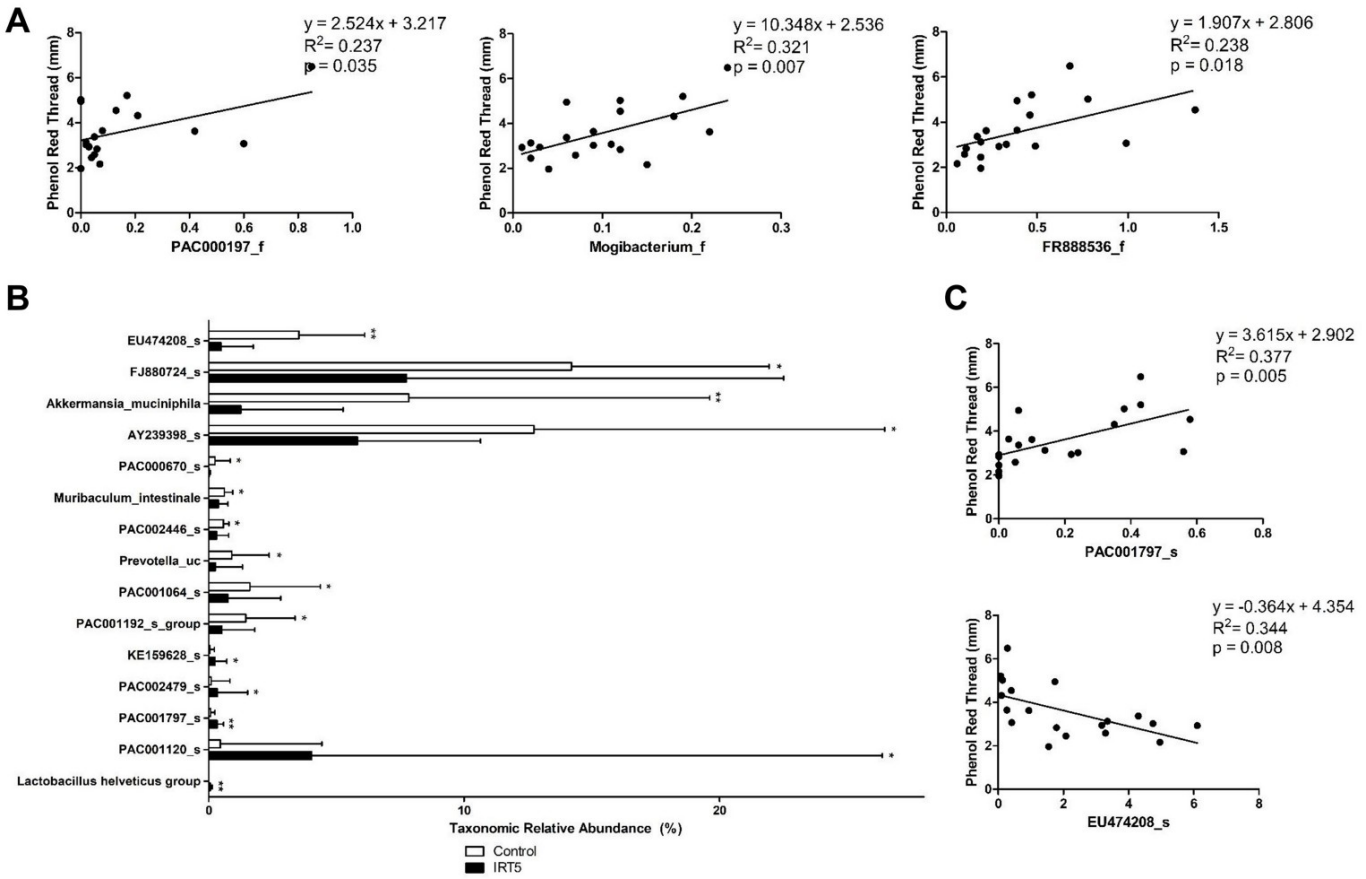
Univariate linear regression analysis of taxons and tear secretion. At the level of family with significant compositional and LEfSE differences, tear secretion showed significant positive association with *Mogibacterium_f* (p = 0.007), which belongs to the order *Clostridiales*, and *FR888536_f* (p = 0.018), which belongs to the phylum *Cyanobacteria* and class *Vampirovibrio_c* (A). Among species with significant compositional differences, including *Lactobacillus helveticus group* (B), tear secretion was positively associated with *PAC001797_s* (p = 0.035), which belongs to the phylum *Cyanobacteria* and class *Vampirovibrio_c*, and inversely related to *EU474208_s* (p = 0.008), which belongs to the family *Muribaculaceae* (C). Statistical analysis with error bars indicating the minimum and maximum data points by Wilcoxon rank-sum test: * p < 0.05, ** p < 0.01.

Univariate linear regression analysis was performed with taxons at the level of family with significant compositional and LEfSE differences, and taxons at the level of species with taxonomic relative abundance average of at least 5% or above (*Lactobacillus helveticus group* was included despite low taxonomic relative abundance because it was the only *Lactobacillus* that had shown significant difference between groups) and significant compositional differences between groups. As a result, tear secretion showed significant positive association with *Mogibacterium_f* (p = 0.007), which belongs to the order *Clostridiales*, and *FR888536_f* (p = 0.018), which belongs to the phylum *Cyanobacteria* and class *Vampirovibrio_c* (Fig 4A). Also among the species that showed significant differences (Fig 4B), tear secretion was positively associated with *PAC001797_s* (p = 0.035), which belongs to the phylum *Cyanobacteria* and class *Vampirovibrio_c*, and inversely related to *EU474208_s* (p = 0.008), which belongs to the family *Muribaculaceae*.

Multivariate linear regression analysis of those variables with p < 0.2 from univariate linear regression analysis was performed in a stepwise manner with adjustment of group classification. As a result, family *Christensenellaceae* (β = -0.608, p = 0.009), and species *Lactobacillus helveticus group* (β = -0.676, p = 0.002) and *PAC001797_s* (β = 0.478, p = 0.011), which belongs to the family *FR888536_f*, order *FR888536_o* and class *Vampirovibrio_c*, and phylum *Cyanobacteria*, were observed to have significant impact on tear secretion.

## Discussion

In the current study, we have demonstrated that IRT5 probiotics treatment partially improves experimental dry eye syndrome by increasing tear secretion which may be associated with the change in intestinal microbiome of experimental dry eye model. This change in tear secretion did not show direct influence from inflammatory regulation in extraorbital lacrimal gland nor cornea and conjunctiva. It suggests that IRT5 treatment possesses only partial effects on environmental dry eye symptoms, while it exhibits more significant effects on autoimmune dry eye symptoms seen in previous studies.

IRT5 group displayed significantly different intestinal microbiome compared to controls. Past studies suggest that reduced intestinal microbiome diversity affects the ocular surface by promoting autoimmunity through the loss of short chain fatty acid (SFCA) producing commensal flora and inducing inflammation [15, 30]. Antibiotics treatment in animal studies observed decrease in intestinal normal flora and diversity which were associated with impairments in the ocular surface that could be reversed with fecal transplantation or probiotics treatment [19, 31, 32]. Allansmith et al., observed that the number of cells containing IgA and IgM in lacrimal glands were reduced in which tear IgA levels were also low in germ-free rats and these levels increased when put under conventional environment [33]. Kudagas et al., found that gut supplementation with *B*. *acidifaciens* increased IgA transcript levels in germ-free mice [31]. These findings suggest the presence of gut-eye-lacrimal gland-microbiome axis which indicates the indirect effects from intestinal microbiome to the eye [16]. In our study, IRT5 probiotics treated group displayed significantly increased intestinal microbiome diversity (Shannon Index, p = 0.041) with different intestinal microbiome compositions (Beta diversity, p = 0.001). Also, IRT5 group demonstrated increased tear secretion (p < 0.001) with significant association with and influence from microbiome changes compared to control.

IRT5 group had relatively increased SFCA-producing bacteria. Fecal analysis showed that IRT5 group had increased *Firmicutes* (p < 0.01) which also resulted with increased F/B ratio compared to control (p < 0.01). Increased F/B ratio is reported to be strongly associated with increased SFCA production because most SFCAs are made by bacteria from the phylum *Firmicutes* [34]. In family, *Ruminococcaceae*, *Lachnospiraceae* and *Christensenellaceae*, which are families belonging to the phylum *Firmicutes*, were also increased in the IRT5 group (p < 0.05). *Ruminococcaceae* and *Lachnospiraceae* are important SFCA and lactic acid producing bacteria [34, 35]. *Ruminococcaceae* is negatively associated with inflammation and is known to regulate lipid profile. Also, some species of *Lachnospiraceae* possess anti-inflammatory properties through butyrate production, one of the main SFCA [36]. *Christensenellaceae*, a ubiquitous micro-organism among animals including human and also a SFCA-producing bacteria, is known to be associated with healthy gut status, longevity and normal body mass index [37]. Though IRT5 is mainly composed of *Lactobacillus species* and despite its increased composition in IRT5 group compared to control, the difference was not significant. This may indicate that the bacteria composing the IRT5 probiotics is not the direct role player in affecting the gut-eye-lacrimal gland-microbiome axis but is more like a coordinator providing an apt environment to encourage beneficial commensal flora growth and action. Also, this minimal increase of *Lactobacilli* may be due to desiccating stress the mice were under in which stress has been reported to be associated with reduction in *Lactobacilli* [38].

Aside from a similarity of increased tear secretion, we have observed some incongruity between experimental dry eye model and Sjögren’s syndrome autoimmune mouse model (NOD.B10.*H2*^*b*^) after IRT5 probiotics treatment [24, 25]. We additionally compared intestinal microbiome of experimental dry eye model treated from the current study to NOD.B10.*H2*^*b*^ mice treated with IRT5 probiotics for 3 weeks without prior antibiotics treatment (S1 Fig). We have observed significant beta diversity difference in both before (S1A Fig, p = 0.001) and after (S1B Fig, p = 0.001) IRT5 treatments. Significant compositional differences were also observed (S1C Fig). There was no difference in the phylum *Firmicutes* (p = 0.463) and *Bacteroidetes* (p = 0.947) nor their ratio (p = 0.739). However, compared to NOD.B10.*H2*^*b*^, experimental dry eye model after IRT5 treatment exhibited increased phylum *Proteobacteria* (p = 0.003), family *Prevotellaceae* (p = 0.006) and *Christensenellaceae* (p = 0.006), and genus *Bacteroides* (p = 0.006) and *Prevotella* (p = 0.009) (S1C Fig). On the contrary, NOD.B10.*H2*^*b*^ after IRT5 treatment revealed increased family *Muribaculaceae* (p = 0.003) and, genus *Bifidobacterium* (p = 0.003) and *Lactobacillus* (p = 0.004) (S1C Fig). NOD.B10.*H2*^*b*^ was seen to have increased *Lactobacillus reuteri group* (p = 0.020), a member of the IRT5 probiotics. Also, NOD.B10.*H2*^*b*^ exhibited increased species *Bifidobacterium pseudolongum group* (p = 0.003), *Lactobacillus gasseri group* (p = 0.003), *Lactobacillus hamster* (p = 0.003), *Lactobacillus helveticus group* (p = 0.014) and *Lactobacillus paracasei group* (p = 0.003). Though NOD.B10.*H2*^*b*^ were treated with IRT5 treatment for 3 weeks, which is longer than the current study of 12 days, this intestinal microbiome difference may be contributed by preexisting genetical difference between mice that creates disparate intestinal environments causing certain species to survive and proliferate while others cannot. However, human clinical studies have also observed intestinal microbiome dissimilarity between Sjögren’s syndrome and non- Sjögren’s syndrome or environmental dry eye subjects, which may indicate the preexistence of distinct intestinal microbiome and environment before disease infliction [17, 39]. Another explanation for the different clinical response to IRT5 treatment between environmental dry eye and Sjögren’s syndrome autoimmune mouse model may be that different immune cells are involved in each disease. While autoimmunity has substantial relation with B cells [40], the intestinal microbiome greatly influences the diversity of B cell clones controlling B cell related chronic inflammations [41, 42]. On the other hand, environmental dry eye disease is an auto-inflammatory disease that is more associated with T cells, such as Th17 or CD4 or CD8 T cells, and so intestinal microbiome influence on B cells may be insufficient to produce significant clinical responses in dry eye diseases [2, 4]. Therefore, the presence of autoimmunity seems to affect the clinical response from IRT5 on dry eye disease.

Several studies have demonstrated the presence of a bidirectional microbiome-gut-brain axis [43–45]. The intestinal microbiome communicates with central nervous system primarily through microbial-derived intermediates that can directly interact with enteroendocrine cells and mucosal immune system, and indirectly affect the nervous system by crossing the intestinal barrier and entering the systemic circulation [38]. Also, several neurotransmitters and neuropeptides, such as neuropeptide Y and substance P, in relation to intestinal microbiome are reported to affect the central and vagal nervous systems [46–48]. Neuropeptide Y, one of the main factors in microbiome-gut-brain axis, may be related with the gut microbiota on inflammatory regulation and brain functions [48]. Also, SFCA, produced by intestinal microbiota, can directly influence the release of peptide YY by enteroendocrine cells [48]. Additionally, depletion of certain intestinal microbiomes solely can directly stimulate vagal neurons and cause firing of sympathetic neurons which can be reversed with fecal transplantation or supplementation of specific microorganisms [45]. The lacrimal gland is innervated by both sympathetic and parasympathetic nerves, and it’s the latter that controls tear secretion [49, 50]. In the present study, only TNF-α in cornea and conjunctiva of IRT5 group increased, while other inflammation related markers did not. Also, additional univariate and multivariate linear regression analysis between goblet cell density and gut microbiome, which were performed in the same manner, revealed inverse correlation with only the species PAC001064_s (p = 0.035), PAC002446_s (p = 0.039) and PAC000670_s (p = 0.041), while there was no significance observed from multivariate linear regression analysis. These may indicate that intestinal microbiome composition in experimental dry eye model indirectly or possibly directly affects the eye via different mechanisms other than regulating inflammation. Change in intestinal microbiome through IRT5 probiotics treatment may subsequently alter the release of certain gut microbial-related neuropeptides, or the compositional change of certain microorganisms itself could affect the parasympathetic component innervating the lacrimal gland to increase tear secretion. In consideration of bacteria forming close relationships with one another and creating a network, multivariate linear regression analysis revealed tear secretion to be strongly influenced by the family *Christensenellaceae* and species *Lactobacillus Helveticus group* and *PAC001797_s*. Family *Christensenellaceae* and species *Lactobacillus Helveticus group* are SFCA-producing bacteria. In particular, species *PAC001797_s* belongs to the phylum *Cyanobacteria*, which is known to accumulate SCFAs under certain conditions through a yet unknown mechanism, and was observed to have significant impact on tear secretion confirmed by both univariate and multivariate linear regression analysis. Therefore, compositional changes of these bacteria may directly influence the parasympathetic nervous system or these bacteria may take part in facilitation of neuropeptides release, such as peptide YY, which subsequently affect the nervous system. Further investigations elucidating this possible mechanism and future studies to discover the properties of specific bacteria are warranted.

There are some limitations to this study. Though the microbiome is known to affect the ocular surface through immunomodulating cells, we did not perform any proteomics study nor other studies regarding changes at cellular level. However, through our previous studies we have already observed that IRT5 probiotics treatment reduces CD8^+^ interferon-γ^hi^ cells and increases regulatory T cells [24]. Also, IRT5 probiotics treatment induces downregulation of proteins associated with defense response and immune system process [25]. Although IRT5 probiotics affects immune system by regulating inflammation, the current study with environmental dry eye exhibits little relevance with inflammation control. Therefore, future studies of probiotics’ mechanism of action should be conducted. In addition, this study was performed using only male mice in order to investigate the probiotics’ effects specifically on environmental dry eye by excluding possible confounding factors such as hormonal effects from female mice. Indeed, dry eye is more common in female subjects, and so future studies regarding probiotics and desiccating stress induced female mice models may be clinically helpful. Also, the number of mice studied in the IRT5 group was relatively smaller than the control group, in which the effects of probiotics might have been more prominent if the IRT5 group had contained more mice. However, with ethical restriction in the number of mice that can be used and in consideration that nine mice is not too small, the IRT5 group in this study still applies as a relative representative of probiotics’ effects. Nevertheless, future studies with a larger group may help elucidate the effects of probiotics that may have been subtle from this study. Also, fecal analysis alone lacks the ability to fully represent the whole intestinal microbiome and microbiome compositions can change according to location of intestinal tract. Another limitation is that the OTU’s were analyzed at a cutoff value of 97%. There may be some microbials sharing more than 97% of entire 16S rRNA. Lastly, we analyzed intestinal microbiome composition, alpha and beta diversities but not their functional properties. The microbiome works in a network manner where one function is not solely dependent on one type of micro-organism but rather several micro-organisms working together to perform that function. Further studies regarding the functional properties of intestinal microbiome and their effects on ocular surface is needed.

## Supporting information

S1 Table Differences in abundance of microbials assessed by LEfSe. (DOCX)

S1 Fig Beta diversity and compositional difference between environmental dry eye C57BL/6 mouse model and NOD mouse model. Beta diversity of genus by UniFrac principle coordinates analysis revealed significant differences before (A) and after (B) IRT5 probiotics treatment (both p = 0.001). Compositional differences of intestinal microbiota after IRT5 probiotics between groups were observed (C). In phylum, *Proteobateria* was increased in environmental dry eye model (p = 0.003) (C). In family, decreased *Muribaculaceae* (p = 0.003) and increased *Prevotellaceae* (p = 0.006) and *Christensenellaceae* (p = 0.006) were observed in environmental dry eye model (C). In genus, decreased proportions of *Bifidobacterium* (p = 0.003) and *Lactobacillus* (p = 0.004) were observed, while *Bacteroides* (p = 0.006) and *Prevotella* (p = 0.009) increased (C). B6: Experimental dry eye model C57BL/6, NOD: Sjögren’s syndrome mouse model (NOD.B10.*H2*^*b*^), P: phylum, F: family, G: genus. Statistical analysis with error bars indicating the minimum and maximum data points by Wilcoxon rank-sum test: ** p < 0.01.
(TIF)
