# Supplementary material for: Effect of IRT5 probiotics on dry eye in the experimental dry eye mouse model
Source: PLoS One. 2020 Dec 1;15(12):e0243176. doi: 10.1371/journal.pone.0243176 (PMC7707591; doi:10.1371/journal.pone.0243176)
Supplement: S1 Table — (DOCX) [file pone.0243176.s001.docx]

| **S1 Table. Differences in Abundance of Microbials Assessed by LEfSe** | | | | | |
| --- | --- | --- | --- | --- | --- |
| **Taxon Name** | **Taxon Rank** | **Control** | **IRT5** | **LDA Effect Size** | **p value** |
| **Increased** |  |  |  |  |  |
| Cyanobacteria | Phylum | 0.21345 | 0.63812 | 3.32730 | 0.00192 |
| Firmicutes | Phylum | 21.41913 | 47.17320 | 5.10982 | 0.00898 |
| Erysipelotrichi | Class | 0.08382 | 0.24542 | 2.90799 | 0.01789 |
| Vampirovibrio_c | Class | 0.21293 | 0.63812 | 3.32783 | 0.00192 |
| Clostridia | Class | 14.50284 | 38.95078 | 5.08722 | 0.00898 |
| Erysipelotrichales | Order | 0.08382 | 0.24542 | 2.90799 | 0.01789 |
| FR888536_o | Order | 0.21293 | 0.63812 | 3.32783 | 0.00192 |
| Clostridiales | Order | 14.50284 | 38.95049 | 5.08721 | 0.00898 |
| Clostridiaceae | Family | 0.04628 | 0.10192 | 2.44869 | 0.02224 |
| Mogibacterium_f | Family | 0.06230 | 0.14836 | 2.63653 | 0.00705 |
| Erysipelotrichaceae | Family | 0.08382 | 0.24542 | 2.90799 | 0.01789 |
| PAC000197_f | Family | 0.03832 | 0.27374 | 3.07149 | 0.03345 |
| FR888536_f | Family | 0.21293 | 0.63812 | 3.32783 | 0.00192 |
| Christensenellaceae | Family | 0.17156 | 0.64033 | 3.37025 | 0.00082 |
| Ruminococcaceae | Family | 5.34587 | 12.88198 | 4.57613 | 0.01789 |
| Lachnospiraceae | Family | 8.79699 | 24.97943 | 4.90802 | 0.01789 |
| PAC001525_g | Genus | 0.00118 | 0.00345 | 2.02262 | 0.04711 |
| PAC001377_g | Genus | 0.00399 | 0.02251 | 2.00982 | 0.00547 |
| PAC001270_g | Genus | 0.00110 | 0.02541 | 2.11351 | 0.00620 |
| PAC001609_g | Genus | 0.00482 | 0.02614 | 2.04761 | 0.00302 |
| PAC001219_g | Genus | 0.01217 | 0.03305 | 2.04940 | 0.04451 |
| PAC002042_g | Genus | 0.01207 | 0.04280 | 2.18949 | 0.04804 |
| JQ084194_g | Genus | 0.00684 | 0.04304 | 2.26681 | 0.02730 |
| PAC001524_g | Genus | 0.00513 | 0.05026 | 2.35696 | 0.00142 |
| PAC001440_g | Genus | 0.01323 | 0.05139 | 2.29827 | 0.01784 |
| PAC000672_g | Genus | 0.01292 | 0.05205 | 2.29931 | 0.03345 |
| Massilioclostridium | Genus | 0.01635 | 0.06074 | 2.35459 | 0.01784 |
| PAC001372_g | Genus | 0.01690 | 0.06703 | 2.40319 | 0.02948 |
| PAC001207_g | Genus | 0.01195 | 0.07369 | 2.49419 | 0.04087 |
| KE159797_g | Genus | 0.01059 | 0.08051 | 2.54536 | 0.00796 |
| Harryflintia | Genus | 0.01362 | 0.08068 | 2.53086 | 0.00061 |
| Arthromitus | Genus | 0.04601 | 0.10068 | 2.44117 | 0.02224 |
| PAC001402_g | Genus | 0.04000 | 0.10705 | 2.53138 | 0.01789 |
| PAC001386_g | Genus | 0.00335 | 0.10960 | 2.72711 | 0.03393 |
| Sporobacter | Genus | 0.06899 | 0.14162 | 2.56508 | 0.01789 |
| PAC001138_g | Genus | 0.03238 | 0.15012 | 2.77136 | 0.01431 |
| Acetatifactor | Genus | 0.03244 | 0.17680 | 2.85915 | 0.02412 |
| PAC000197_f_uc | Genus | 0.00474 | 0.17708 | 2.93635 | 0.02307 |
| PAC001360_g | Genus | 0.05199 | 0.24266 | 2.97976 | 0.00082 |
| Agathobaculum | Genus | 0.04161 | 0.26270 | 3.04400 | 0.02468 |
| PAC001199_g | Genus | 0.08380 | 0.31337 | 3.06029 | 0.00548 |
| Clostridium_g24 | Genus | 0.07475 | 0.31952 | 3.08810 | 0.00550 |
| Alloprevotella | Genus | 0.10236 | 0.33310 | 3.06269 | 0.03221 |
| Anaerotruncus | Genus | 0.11007 | 0.46174 | 3.24536 | 0.03376 |
| FR888536_g | Genus | 0.21293 | 0.63812 | 3.32783 | 0.00192 |
| PAC001092_g | Genus | 0.25754 | 0.99655 | 3.56777 | 0.02224 |
| Pseudoflavonifractor | Genus | 0.46942 | 1.43934 | 3.68581 | 0.01137 |
| LLKB_g | Genus | 0.23776 | 1.48360 | 3.79450 | 0.01784 |
| PAC000664_g | Genus | 0.56082 | 1.57273 | 3.70420 | 0.03376 |
| PAC001525_s | Species | 0.00118 | 0.00345 | 2.02262 | 0.04711 |
| PAC001743_s | Species | 0.00029 | 0.00411 | 2.07344 | 0.00093 |
| PAC001070_s | Species | 0.00739 | 0.01408 | 2.03992 | 0.03725 |
| PAC001377_s | Species | 0.00399 | 0.02251 | 2.00982 | 0.00547 |
| EU772178_s | Species | 0.00249 | 0.02580 | 2.09701 | 0.01812 |
| EU511112_s | Species | 0.00471 | 0.02587 | 2.08624 | 0.00338 |
| PAC001713_s | Species | 0.00371 | 0.02694 | 2.10685 | 0.00132 |
| AB622833_s | Species | 0.00279 | 0.02862 | 2.11894 | 0.00448 |
| PAC001740_s | Species | 0.00659 | 0.02929 | 2.08108 | 0.00796 |
| PAC001369_s | Species | 0.00607 | 0.02958 | 2.11277 | 0.00251 |
| PAC001801_s | Species | 0.00251 | 0.03075 | 2.15858 | 0.04698 |
| PAC001785_s | Species | 0.00000 | 0.03205 | 2.20867 | 0.00846 |
| PAC001557_s | Species | 0.00886 | 0.03299 | 2.12055 | 0.01442 |
| JQ084476_s | Species | 0.00426 | 0.03330 | 2.17125 | 0.01231 |
| PAC002042_s | Species | 0.00595 | 0.03365 | 2.14833 | 0.03052 |
| PAC001360_g_uc | Species | 0.00497 | 0.03482 | 2.19100 | 0.02797 |
| FR888536_g_uc | Species | 0.00862 | 0.03558 | 2.14653 | 0.02797 |
| PAC001560_s | Species | 0.00308 | 0.03586 | 2.22565 | 0.00077 |
| PAC000183_s | Species | 0.00371 | 0.03827 | 2.24671 | 0.00547 |
| Neglecta timonensis | Species | 0.00275 | 0.03861 | 2.26403 | 0.03830 |
| PAC001547_s | Species | 0.01328 | 0.03896 | 2.13674 | 0.02749 |
| PAC001518_s | Species | 0.01476 | 0.03902 | 2.10205 | 0.02218 |
| EU455092_s | Species | 0.00667 | 0.04078 | 2.24648 | 0.00142 |
| PAC001574_s group | Species | 0.00994 | 0.04176 | 2.21138 | 0.03650 |
| JQ084194_s | Species | 0.00478 | 0.04193 | 2.27752 | 0.01046 |
| PAC001131_s | Species | 0.01046 | 0.04471 | 2.25249 | 0.00695 |
| PAC001742_s | Species | 0.01176 | 0.04668 | 2.24596 | 0.02224 |
| PAC002453_s | Species | 0.00719 | 0.04827 | 2.31935 | 0.01346 |
| PAC001524_s | Species | 0.00485 | 0.04877 | 2.34531 | 0.00142 |
| PAC001371_s | Species | 0.01433 | 0.05328 | 2.29221 | 0.00427 |
| Lactobacillus helveticus group | Species | 0.00131 | 0.05526 | 2.44171 | 0.00019 |
| PAC001222_s | Species | 0.00349 | 0.05926 | 2.44848 | 0.00215 |
| PAC001521_s | Species | 0.01635 | 0.06043 | 2.35151 | 0.01784 |
| PAC001372_s | Species | 0.01377 | 0.06217 | 2.38781 | 0.03632 |
| AB606300_s | Species | 0.00167 | 0.06437 | 2.49928 | 0.01242 |
| PAC001109_g_uc | Species | 0.00384 | 0.06526 | 2.49545 | 0.04711 |
| PAC001746_s | Species | 0.01310 | 0.06602 | 2.42690 | 0.03299 |
| PAC002505_s | Species | 0.01136 | 0.07733 | 2.52039 | 0.00425 |
| PAC001549_s | Species | 0.00881 | 0.07825 | 2.54356 | 0.04451 |
| PAC001366_s | Species | 0.02072 | 0.08989 | 2.54016 | 0.03221 |
| DQ777929_s | Species | 0.01797 | 0.09289 | 2.57602 | 0.00703 |
| AP012202_s group | Species | 0.04601 | 0.10068 | 2.44117 | 0.02224 |
| Pseudoflavonifractor_uc | Species | 0.03337 | 0.10306 | 2.54478 | 0.04123 |
| PAC002391_s | Species | 0.02341 | 0.10610 | 2.61837 | 0.02681 |
| PAC001386_s | Species | 0.00335 | 0.10960 | 2.72711 | 0.03393 |
| PAC002511_s group | Species | 0.02425 | 0.12523 | 2.70505 | 0.04087 |
| Flintibacter butyricus group | Species | 0.03545 | 0.12924 | 2.67305 | 0.02749 |
| PAC001501_s | Species | 0.02083 | 0.15446 | 2.82579 | 0.01784 |
| PAC001186_s group | Species | 0.04174 | 0.15636 | 2.75908 | 0.02224 |
| PAC001925_s | Species | 0.01626 | 0.16201 | 2.86339 | 0.03041 |
| PAC001083_s | Species | 0.03858 | 0.16790 | 2.81137 | 0.00961 |
| PAC001374_s | Species | 0.02452 | 0.17386 | 2.87392 | 0.00542 |
| KI535319_s | Species | 0.05016 | 0.17931 | 2.81089 | 0.02749 |
| PAC001540_s | Species | 0.02586 | 0.18988 | 2.91482 | 0.04964 |
| KE159628_s | Species | 0.04247 | 0.24264 | 3.00089 | 0.01133 |
| PAC001797_s | Species | 0.07099 | 0.32413 | 3.10274 | 0.00656 |
| PAC002479_s | Species | 0.10236 | 0.33310 | 3.06269 | 0.03221 |
| PAC002428_s | Species | 0.00000 | 0.54749 | 3.43752 | 0.00846 |
| KE159605_s | Species | 0.28754 | 1.21172 | 3.66484 | 0.04904 |
| AB606236_s | Species | 0.09954 | 1.25150 | 3.76049 | 0.03658 |
| PAC001120_s | Species | 0.45587 | 4.02073 | 4.25104 | 0.03299 |
| **Decreased** |  |  |  |  |  |
| Saccharibacteria_TM7 | Phylum | 1.45117 | 0.52054 | 3.66785 | 0.03376 |
| Verrucomicrobia | Phylum | 7.83707 | 1.25239 | 4.51752 | 0.00898 |
| Bacteroidetes | Phylum | 62.26908 | 41.86771 | 5.00863 | 0.01137 |
| Saccharimonas_c | Class | 1.45117 | 0.52054 | 3.66785 | 0.03376 |
| Verrucomicrobiae | Class | 7.83707 | 1.25239 | 4.51752 | 0.00898 |
| Bacteroidia | Class | 62.23134 | 41.77948 | 5.00971 | 0.01137 |
| Saccharimonas_o | Order | 1.45117 | 0.52054 | 3.66785 | 0.03376 |
| Verrucomicrobiales | Order | 7.83707 | 1.25224 | 4.51753 | 0.00898 |
| Bacteroidales | Order | 62.23134 | 41.77948 | 5.00971 | 0.01137 |
| Saccharimonas_f | Family | 1.45117 | 0.52054 | 3.66785 | 0.03376 |
| Akkermansiaceae | Family | 7.83707 | 1.25224 | 4.51753 | 0.00898 |
| Prevotellaceae | Family | 34.37106 | 19.35734 | 4.87546 | 0.01431 |
| PAC002448_g | Genus | 0.02109 | 0.00086 | 2.12992 | 0.00016 |
| PAC001097_g | Genus | 0.20915 | 0.00090 | 3.01843 | 0.01093 |
| Rikenella | Genus | 0.37786 | 0.03747 | 3.23166 | 0.03345 |
| Muribaculaceae_uc | Genus | 0.27647 | 0.11905 | 2.89845 | 0.02749 |
| PAC001066_g | Genus | 0.41938 | 0.20502 | 3.03073 | 0.04123 |
| PAC001692_g | Genus | 0.62005 | 0.31384 | 3.18607 | 0.01137 |
| PAC001112_g | Genus | 0.72164 | 0.37758 | 3.23612 | 0.00550 |
| PAC000677_g | Genus | 1.45117 | 0.52054 | 3.66785 | 0.03376 |
| Akkermansia | Genus | 7.83707 | 1.25224 | 4.51753 | 0.00898 |
| Prevotella | Genus | 18.00512 | 10.35555 | 4.58262 | 0.04123 |
| PAC000186_g | Genus | 7.80951 | 3.71838 | 4.31084 | 0.01137 |
| Paraprevotella | Genus | 14.20985 | 7.74434 | 4.50959 | 0.04123 |
| PAC001122_s | Species | 0.21735 | 0.00000 | 3.03656 | 0.00754 |
| PAC001097_s | Species | 0.20888 | 0.00000 | 3.01973 | 0.00754 |
| PAC001678_s | Species | 0.00273 | 0.00000 | 2.02140 | 0.01779 |
| PAC001127_g_uc | Species | 0.00456 | 0.00000 | 2.12001 | 0.03935 |
| PAC002009_s group | Species | 0.05664 | 0.00016 | 2.45442 | 0.04779 |
| EU791023_s | Species | 0.03208 | 0.00024 | 2.21482 | 0.00055 |
| AB606390_s | Species | 0.01176 | 0.00086 | 2.17405 | 0.00081 |
| PAC001063_g_uc | Species | 0.00710 | 0.00103 | 2.14413 | 0.00745 |
| AM265449_s | Species | 0.03434 | 0.00962 | 2.11676 | 0.00656 |
| Rikenella_uc | Species | 0.13128 | 0.01287 | 2.77697 | 0.01346 |
| PAC000670_s | Species | 0.24658 | 0.02460 | 3.04673 | 0.04087 |
| PAC001267_s | Species | 0.07804 | 0.03255 | 2.36234 | 0.01789 |
| PAC001359_s | Species | 0.10696 | 0.04806 | 2.47640 | 0.01789 |
| EU622763_s | Species | 0.20781 | 0.05675 | 2.87883 | 0.01123 |
| PAC002452_s | Species | 0.18435 | 0.12669 | 2.46664 | 0.04123 |
| PAC001075_s | Species | 0.44461 | 0.24771 | 2.99619 | 0.02224 |
| Prevotella_uc | Species | 0.90398 | 0.25348 | 3.51242 | 0.01431 |
| PAC002446_s | Species | 0.57828 | 0.30332 | 3.13943 | 0.01789 |
| Muribaculum intestinale | Species | 0.60838 | 0.37439 | 3.06876 | 0.02749 |
| EU474208_s | Species | 3.53634 | 0.48182 | 4.18397 | 0.00033 |
| PAC001192_s group | Species | 1.44297 | 0.51294 | 3.66758 | 0.03376 |
| PAC001064_s | Species | 1.61391 | 0.75363 | 3.63374 | 0.03376 |
| Akkermansia muciniphila | Species | 7.83625 | 1.25224 | 4.51747 | 0.00898 |
| AY239398_s | Species | 12.73321 | 5.82063 | 4.53862 | 0.01137 |
| FJ880724_s | Species | 14.20353 | 7.74176 | 4.50934 | 0.04123 |
