# Supplementary figures and images for: Effect of IRT5 probiotics on dry eye in the experimental dry eye mouse model
Source: PLoS One. 2020 Dec 1;15(12):e0243176. doi: 10.1371/journal.pone.0243176 (PMC7707591; doi:10.1371/journal.pone.0243176)

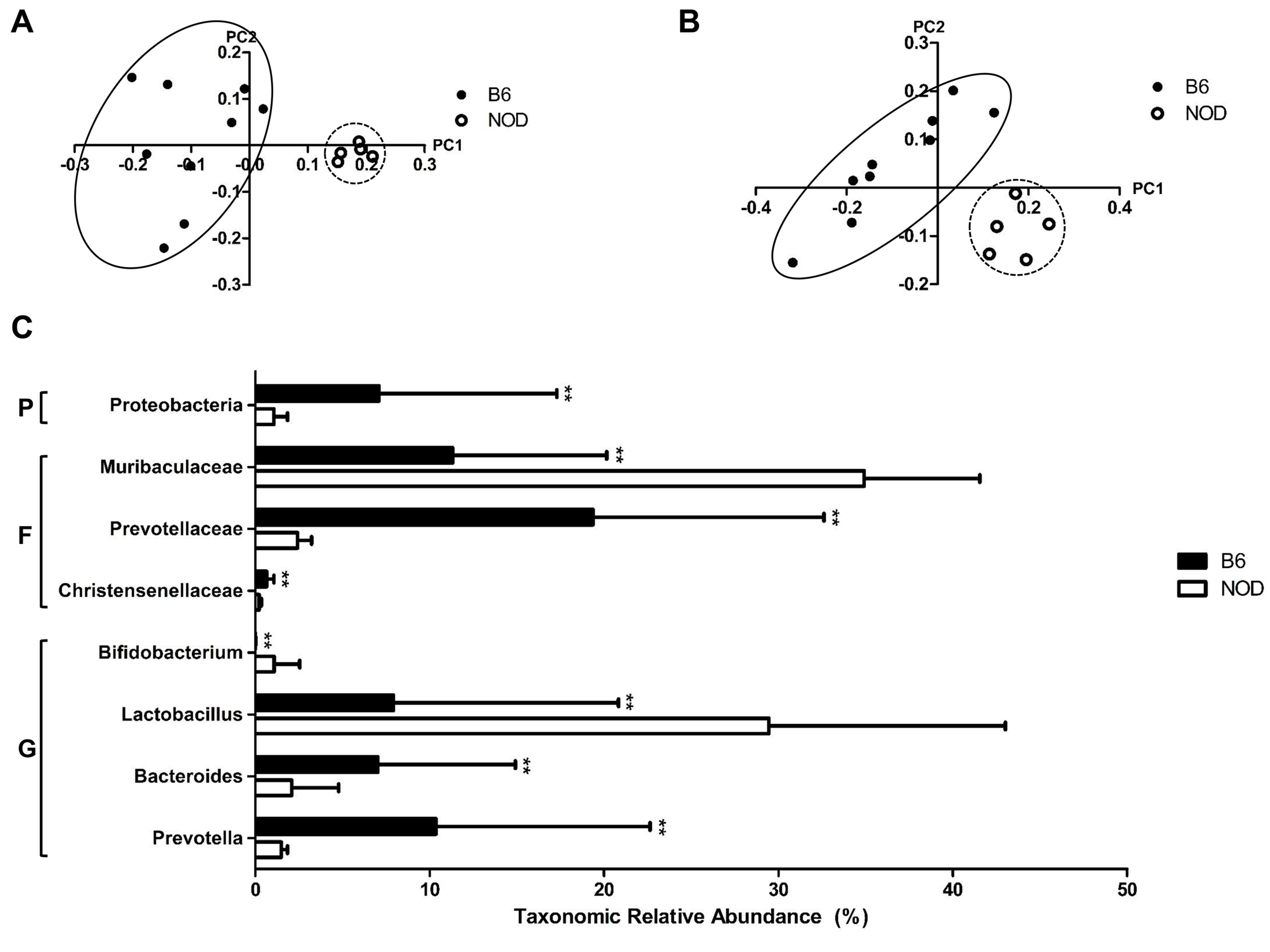

Supplement: S1 Fig — Beta diversity of genus by UniFrac principle coordinates analysis revealed significant differences before (A) and after (B) IRT5 probiotics treatment (both p = 0.001). Compositional differences of intestinal microbiota after IRT5 probiotics between groups were observed (C). In phylum, Proteobateria was increased in environmental dry eye model (p = 0.003) (C). In family, decreased Muribaculaceae (p = 0.003) and increased Prevotellaceae (p = 0.006) and Christensenellaceae (p = 0.006) were observed in environmental dry eye model (C). In genus, decreased proportions of Bifidobacterium (p = 0.003) and Lactobacillus (p = 0.004) were observed, while Bacteroides (p = 0.006) and Prevotella (p = 0.009) increased (C). B6: Experimental dry eye model C57BL/6, NOD: Sjögren’s syndrome mouse model (NOD.B10.H2b), P: phylum, F: family, G: genus. Statistical analysis with error bars indicating the minimum and maximum data points by Wilcoxon rank-sum test: ** p < 0.01. (TIF) [file pone.0243176.s002.tif]
